# 

**Published:** 1877-05

**Authors:** 


### THE AMERICAN JOURNAL OF DENTAL SCIENCE.

EDITED BY

F. J. S. GORGAS, M. D., D. D. S.,

AND

JAMES B. HODGKIN, D. D. S.

---

VOL. 11.—THIRD SERIES.

SURGEON GEN'LS OFFICE
75266
LIBRARY.

---

BALTIMORE:

SNOWDEN & COWMAN.

LONDON:

TRUBNER & CO., 60 PATERNOSTER ROW.

1877 — ~~1878~~.

### INDEX TO VOLUME XI.---THIRD SERIES.

A Case in Practice, - - - - - - 333
A New Counter Irritant - - - - 572
A New Dental Inhaler, - - - - - 87
A New Method of Pivoting Teeth, - - - 280
A New Degree in Dentistry, - - - - 278
A New Dental Quarterly, - - - - 276
A Young Mother, - - - - - 48
A Secret Pile Remedy, - - - - 47
A Simple Cautery, - - - - - 85
A Simple Telephone, - - - - 573
A Plea for Life Assurance, - - - - 385
A Daring Therapeutist, - - - - - 432
A Question of Articulation, - - - - 379
Adenoma of Soft Palate, - - - - 37
Advice to Students, - - - - - - 96
American Dental Association, - - - 53, 193
American Dentists in Europe, - - - 574
American Academy of Dental Surgery, - - 1
Anomalies in Digestion, - - - - 83
An Iron Calculus, - - - - - - 96
Anæsthesia, The Discovery of, - - - 55
Anæsthesia, - - - - - - 188
Anæsthesia, Local, - - - - - 189
Astringents upon the Vessels, Local Action of, - 47
Atmospheric Anæsthesia, - - - - - 39
Adænoid Tumor of Soft Palate, - - - 133
Action of Anæsthetics upon Muscles and Nerves, - 140
An Antidote to Chloroform, - - - - 144
Address of President of Virginia State Dental Association, 433
Acquittal of Dr. Warren C. Westlake, in the Chloroform Case, 92
Anderson, C. L., M. D., - - - - - 221
Apthæ, - - - - - - - 237
A Few Thoughts on Treatment of Lower Biscuspid Teeth, 354
Abscess of Antrum, - - - - - 379
Alumni Meeting, - - - - - 421, 477 479
American Medical Diplomas Abroad, - - 382
Alumni Association of Baltimore College of Dental Surgery, 421

Action of the Nervous System, - - - 289
Arsenic in Vulcanite, - - - - - 427
Atheroma, - - - - - - - 431
Anæsthetic Effects of Cold Water, - - - 333
Animal Magnetism, - - - - - 336
Anæsthetics, Effects of Human System as evidenced by
Spectroscopic Observations, - - - 481
Anæsthetics, What Shall We Use, - - - 505
Arsenic and Antimony in Vulcanized Rubber, - 527
Advertisng Dentists, - - - - 333
Alumni Meeting of B. C. D. Surgery, - - - 479
American Dental Convention, - - - 167, 241
Anæsthesia, Local, by Bromhydrate of Quinine, - 189
Alveolar Abscess, - - - 226
Acidity of the Gastric Juice in Man, - - 238
Action of Nitrite of Amyl, - - - - 275
Anæsthetics, Unusual Action of, - - - 522
Barrett, W. C., D. D. S., - - - - 464
Barker, Prof. Geo. T., D. D. S., M. D., - - - 31
Blue Glass, - - - - - 95, 474
Brown, N. F., M. D., - - - - - 125
Bromide of Potassium as a Caustic, - - - 189
Beale, Lionel S., - - - - - 381
BIBLIOGRAPHICAL.
Taft's Operative Dentistry, Third Edition, - - - 44
Harris' Medical and Dental Dictionary, Fourth Edition, 425
Biddle's Materia Medica, Eighth Edition, - - 426
Elements of Dental Materia Medica and Therapeutics, 426
Scientific American, - - - - - 432
American Agriculturist, - - - - - 432
Vicks' Illustrated Catalogue for 1878, - - 475
Music Publications, - - - - - 475
Contributions to History of Medical Education & Medical Institutions of U. S. of America, 1776 to 1876, 476
Wine in the Different Forms of Anæmic and Gout, - 476
A Review of the Recent Theories of Nerve Action, the Use of Electricity in Medicine and Surgery, &c., 476
Tanner's Index of Disease and their Treatment. - 283
The Physicians' Visiting List for 1878, - - 283
The Transactions of American Dental Association 1876, 283
The Transactions of Illinois State Dental Society for 1877, - - - - - - - 284
The Dental Office and Laboratory, - - 136
Harris' Medical and Dental Dictionary, A new Edition, 331
Bichromate of Potash as an Antiseptic, - - - 430
Broaches for Pulp Canals, - - - - - 332
Carbolized Camphor, Therapeutic Action of, - - 48
Causes of Failures in the Use of Cohesive Gold Foil, the Mallet, &c., - - - - - - 22

Cleborne, C. J., M. D., - - - - - 87
Comparative Anatomy, - - - - 40
Congestion of the Pulp, - - - - - - 31
Chloral Hydrate, Local Action of, - - - 92
Croton Chloral in Dentistry, - - - 87, 236
Carbolized Camphor, - - - - - 93
Celluloid vs. Rubber, - - - - - - 94
Cautery, - - - - - - 85
Chloroform. Dangers of, - - - - - - 121
Case of Exsection of Half of Inferior Maxillary Bone,
Reproduction of Bone, - - - - - 129
Chloroform, Death from, - - - - 140, 191
Croton Chloral in Facial Neuralgia, - - - 141
Chlorate of Potash and Mercurials, - - - 143
Chloroform, Mode of Administering, - - 143
Cold in the Head, How to Cure, - - - - 144
Chloroform, Death from Self-Administration of, - 144
Campbell, Dr. J., - - - - 157
Cloudiness on Exploring Mirrors, Method of Preventing, 144
Chloral, Death from Ten Grains of, - - - 142
Chase, Dr. Henry S. and his Theories, - - - 157
Carbolic Acid for Neuralgia, Injection of, - - 188
Camphor, Phenicated, - - - - - - 190
Chase, Henry S., M. D., D. D. S., - - - 179
Canadian Dentist in Germany, - - - - 206
Cold Bath in Infantile Diarrhoea, - - - 234
Carbolic Acid in Diarrhoea, - - - - 235
Cold, Therapeutic Use of, - - - - 239
Chloroform and Ether in their Medico-Legal Relations, 229
Caldwell, John J., M. D., - - - 289, 348, 359
Connection between Excessive Brain worry and Bodily
Disease, - - - - - - - 359
Celluloid Case, Decision in the, - - - 374
Composition of Sozodont, &c, - - - - 378
Chloral Hydrate, External Use of, - - - 382
Chloral in Tetanus, - - - - - - 384
Cleft Palates, Essay on, - - - - 411
Celluloid, Preparation of, - - - - - 428
Colored Borax Varnishes, - - - - 428
Capping Pulp, - - - - - - - 464
Chancre of the Lip, - - - - - 468
Cause of Dental Caries, - - - - - - 471
Chloroform, Antidote to, - - - - 144
Celluloid, - - - - - - - 519
Chloral for Removing warts, - - - - 523
Curtis, Lester, M. D., - - - - - - 322
Chloroform, Subcutaneous Injections of, - - 525
Chloral, Can Twenty Grains Kill? - - - 526

Caries, Disentegration, &c., of, - - - - 107
Chisholm, Prof. Julian J., M. D., - - - - - 505
Chemistry for Dental Students. - - - - 568
Cyanide of Zinc in Facial Neuralgia, - - - 576
Calculus in the Sublingual Duct, - - - - 573
Circassian Surgery, - - - - - - 527
Chloroform Hallucination and Alleged Rape, - - 528
Death from Self-Administration of Chloroform, - 144
Death from Chloroform, - - - - - - 140
Death from Chloral, - - - - - 142
Death from Nitrous Oxide Gas and Ether, - - - 89
Death from Nitrous Oxide, - - - - - 49
Death of Dr. Charles A. Jourdain from Chloroform, - 6
Dislocation of Molar Bone, - - - - - 46
Dental Association Meetings, - - 52, 53, 54, 138
Discovery of Anæsthesia, - - - - - 55, 114
Dislocation of Gold Plugs, - - - - - 78
Dental Inhaler, - - - - - - - 87
Digestion, Anomalies in, - - - - - - 83
Diseased Gums, - - - - - - 97
Disintegration, Arrestation and Prevention of Caries, - 107
Dangers of Chloroform, - - - - - 121
Dyspepsia, - - - - - - - 134
Donation from Prof. Henry S. Chase, M. D., D. D. S., - 138
Diseased Conditions, The Principles involved in the Treatment of, - - - - - - - 145
Dr. Chase, and his Theories, - - - - 157
Death while under Ether, - - - - - - 178
Dental Alloys, - - - - - - 179
Death During Etherization, - - - - 190
Denig, Dr. A. M., - - - - - - 229
Death from Anæsthetics, - - - - - - 236
Dangerous Prescriptions, - - - - 240
Dr. Edward Warren, - - - - - - - 240
Development of Dentine, - - - - 298
Decline of Mechanical Dentistry, - - - 328, 334
Davyum, - - - - - - - 334
Diverse and Divers Views, - - - - - - 335
Degeneration of the Tongue and Tissues at its Base, - 337
Decision in the Celluloid Case, - - - - 374
Dangers of Ether, - - - - - - 383, 429
Dr. Davenport, - - - - - - - 411
Dental Education as seen by a Canada Editor, - - 422
Dental Caries, Causes of, - - - - - - - 471
Death from Chloroform Averted by Nitrite of Amyl, 477, 525
Death from Inhalation of Ether, - - - - - 479
Dr. Waterman on Spectroscopic Analysis, - - - 518
Deaths from Anæsthetics in 1877, - - - - - 526

District of Columbia and Maryland Dental Society, - 241
Dental Mechanism, Practical Hints in, - - - - 332
Danger of Ether, - - - - - - - - 571
Death from a Carious Tooth, - - - - - 573
Death from Chewing Tobacco, - - - - - - 575
Exsection of Half of Inferior Maxillary, Reproduction of Bone, - - - - - - - 129
Exploring Mirrors, Method to Prevent Cloudiness of, - 144
Etiology of Alcoholic Degeneration, - - - - 170
Excision of Inferior Maxillary, - - - - - 185
Excision of Branches of Fifth Pair of Nerves for Neuralgia, 186
Editorial Briefs, - - - - - - 281
Eucalyptus as a Local Anæsthetic, - - - 335
Efficacy of Electricity in Dental Surgery. - - - 337
External Use of Chloral Hydrate, - - - - 382
Essay on Cleft Palates, - - - - - - 411
Excision of Inferior Dental and Gustatory Nerves for Cure of Dental Neuralgia, - - - - - - 479
Effect of Anæsthetics upon the Human System as evidenced by Spectroscopic Observations, - - - - 481
Eley, M. J., M. D., - - - - - - 39
English Dentistry, Status of, - - - - - 381
Functions of the Liver, - - - - - 428
Flagg, J. Foster, D. D. S., - - - - 419
Filling Teeth with Gold Foil, - - - 164
Farquharson, Dr., - - - - - 134
Fractures, Phosphate of Lime in Relation to, - 427
Gastric Juice, Properties of, - - - - 192
Gold Plugs, Discoloration of, - - - 78
Gibbons, H., M. D., - - - - - - 69
Good Teeth, How to have, - - - - 372
Gold, Substance of Lectures on, - - - 511
General Nervousness in the Male - - - 524
Gingivitis—Its Different Forms, &c., - - - 547
How to Have Good Teeth, - - - - - 372
How to Cure a Cold in the Head, - - - 144
How to Deprive Iodine of its Stain, - - - 141
Homœopathy in Danger, - - - - - - 184
Hodgkin, Prof. Jas. B., D. D. S., - 276, 343, 385, 511
Hydrobromic Ether as an Anæsthetic, - - 286
Harris' Medical and Dental Dictionary, A New Edition, 331
Hand-Pressure and Soft-Foil, vs. Malleting and Cohesive Gold, - - - - - - - - 419
Harlan, Dr. A. W., - - - - - 354
Indigestible Substances, - - - - - 91
Iron Calculus, - - - - - - 96
Ingersoll, L. C., D. D. S., - - - - - 97
Injection of Carbolic Acid for Neuralgia, - - - 188

India Rubber Cloth in Diseases of the Skin, - - - 237
Inhibition of Publication and its Result, - - 282
Iron Cement, - - - - - 528
Is the Human Eye Changing its Form, &c, - - 570
Injury from Disturbed Sleep, - - - 572
Jottings in Operative Dentistry, - - - 459
Local Action of Astringents upon the Vessels, - - 47
Local Action of Chloral Hydrate, - - - 92
Local Anæsthesia, - - - - - - 189
Literary Estimate of the Medical Art, - - 127
Length of the Frænum of the Tongue, - - - 187
Letter from Oakland, - - - - 232
Lacto-Phosphate of Lime as a Tooth Filling, - - 238
Lepoma of Tongue, - - - - 287
Limits of the Optical Capacity of the Microscope, - 322
Letter Concerning the Southern Dental Association, 330
Lecture, Outline of a, - - - - - 343
Liquefaction of Gases, - - - - 523
Lower Bicuspids, Treatment of, - - - 354
Magnetism, Animal, - - - - - .336
Meeting of Dental Associations, - - - 138
Method of Preventing Cloudiness of Exploring Mirrors, 144
Metallotheraphy, - - - - - 173
Medical Examinations in Scotland, - - 142
Medico-Legal Relations of Chloroform and Ether, - 229
Mechanical Dentistry, - - - - 262
Maryland and District of Columbia Dental Society, - 241
Microscope, The—Its Misinterpretations, - - 271
Medical Progress, - - - - - - 282
Menstruation, Rest During, - - - 284
McQuillen, Prof. J. H., D. D. S., M. D., - - - 314
Mechanism, Dental, - - - - 332
Malaria, - - - - - - 336
Mixed Narcosis, - - - - 576
Michigan Dental Association, - - - - 131
Making Rubber Stamps, - - - - 384
McGraw, Theodore, M. D., - - - - 364
Mastic for Fastening Rubber on Metals, - - 429
McLean, Robert, M. D., - - - - - 129
Mitchell, S. Weir, M. D., - - - - 398
Nitrous Oxide Gas as an Anæsthetic, - - - 69
Nitrous Oxide Gas, Death from, - - - 49
New Dental Inhaler, - - - - - - 87
Nelaton's Method of Resuscitation from Chloroform, 142
North Carolina State Dental Association, - - 181, 262
Nussbaum's Narcosis, - - - - - - 191
New Method of Pivoting Teeth, - - - 278
New Treatment of Plaster Casts, - - - - 397

Nervousness in the Male, - - - - - 398
Nitrite of Amyl, - - - - - - 431
Neuralgia Treated with Blue-Glass, - - - 474
Nitric Acid for Hoarseness, - - - - 480
New Anæsthetic, - - - - - - 574
OBITUARY NOTICES.
Dr. William D. Jenks, - - - - 45
Thos. De Graffenreid, D. D. S., - - 139
Prof. Henry Reginald Noel, M. D., - - - 472
Prof. George T. Barker, D. D. S., - - 520
John Ensor La Motte, D. D. S., - - - 522
Dr. George J. Barclay, D. D. S., - - 569
On Congestion of the Pulp - - - - 31
On Dyspepsia, - - - - - 134
Osseous Lesions in Infantile Syphilis, - - - 239
On Ranula, - - - - - 286
Outline of a Lecture, - - - - - 343
Observations on Articulation of Artificial Teeth, - 368
Palmer, S. B., D. D. S., - - - - - 164
Peabody, F., M. D., - - - - - - 145
Pennsylvania State Dental Society, - - - 53
Possible Danger from Salicylic Acid, - - 39
Preliminary Education of Medical Students, - - 95
Precautions in Using the Hypodermic Syringe, - 139
Principles Involved in the Treatment of Diseased Conditions, 145
Periosteal Surgery in the United States, - - - 176
Phenicated Camphor, - - - - - 190
Poisonous Properties of Glycerine, - - - 190
Properties of the Human Gastric Juice, - - - 192
Plastic Fillings, - - - - - - 221
Perils of the Cigar, - - - - - - 285
Percussion of Bones, - - - - - - - 286
Present Number of Journal, - - - 331
Practical Hints in Dental Mechanism, - - - 332
Prince, David, M. D., - - - - 337
Plaster Casts, New Treatment of, - - - 397
Phosphate of Lime in Fractures, - - - 427
Pulp Capping, - - - - - - 464
Plastic Filling, and the Basal Principles of the New Departure, - - - - - - - 556
Plating of Iron and Steel with Nickel and Cobalt by Immersion, - - - - - - 476
Principles Involved in Filling Teeth with Gold Foil, - 164
Radical Cure of Neuralgia of Inferior Dental Nerve, - 81
Rubber vs. Celluloid, - - - - - - 94
Reproduction of Bone, - - - - - 129
Russian Female Medical School, - - - - - 192
Remarks of Prof. Huxley to Dental Students, - 215

Rohland, Dr. C. B., - - - - - - 262
Rest During Menstruation, - - - - - 284
Repeated Excision of Inferior Dental and Gustatory Nerves
for Cure of Neuralgia, - - - 364, 479
Results of Intermarriage, - - - - - 574
Removing Salivary Calculus from Sublingual Gland, - 380
Relief for Foreign Bodies in the Ear, - - 571
Salicylic Acid Bad for the Teeth, - - - 237
Salicylic Acid and its Compounds, - - - 141
Salicylic Acid, Possible Dangers from, - - - 39
Salicylic Acid, Use of, - - - - - 55
South Carolina State Dental Association, - - 52
Southern Dental Association, - - - 54, 167, 241
Sub-Luxation of Inferior Maxillary Backwards, - 75
Solvent for Rubber, - - - - - 91
Symptoms in Young Patients from Anæsthesia by Ether, 91
Stimulants Used by the Race, - - - - 96
Shaw, Dr. S. Parsons, - - - - 49
Sims, J. Marion, M D., - - - 55, 114
Sub-Periosteal Surgery, - - - - 135
Suppression of the Salivary Secretion, - - - 191
Scientific Theories vs. Observed Facts, - - 257
Simple Remedy to Stop Bleeding, - - - 285
Secondary Dentine, - - - - 336
Sihler, C., M. D., - - - - - 298
Scirrhus and Epithelial Degeneration of the Tongue, - 337
Superlative Velocity of Electricity, - - - 348
Sozodont, Composition of, - - - - 378
Simple Method of Testing the Purity of Chloroform, - 381
Specific for Diptheria, - - - - 427
Salicylic Acid and Borax, - - - 430
Spectroscopic Analysis, - - - - 518
Supra-Orbital Neuralgia, - - - - 524
Substance of Lectures, on Gold, - - - 511
Subcataneous Injections of Chloroform, - - - 525
Susquehanna Dental Association, - - - 7
Soft Palate, Adenoma of, - - - - - 37
Secret Pile Remedy, - - - - - 46
Strychnia as a Substitute for Quinia - - - 335
Should Five Years Dental Practice be Equivalent to a
Course of Lectures, - - - - - 314
Taylor, W. W., M. D., - - - - - 133
Tennessee Dental Association, - - - - - 54
To Take Rust out of Steel, - - - - - 429
Therapeutic Use of Gold, - - - - 239
Tetanus, - - - - - - 125
The Johns Hopkins Hospital, - - - - 182
The Treatment of Atheromatous Cysts of the Neck, - 187

The Length of Frænum of Tongue, - - - 187
Turner, Dr. J. A., - - - - - 226
The Odors of Persons, - - - - 526
The Effect of Anæsthetics upon the Human System as Evidenced by Spectroscopic Observations, - - 481
The Use of the Mallet and Cohesive Gold Foil, - 22
The So-called Riggs' Disease and its Treatment, - - 563
The Thirty-Eighth Annual Commencement of the Baltimore College of Dental Surgery, - - - 567
The Dental and Oral Science Magazine, - - - 569
The Purity of Chloral, - - - - 575
Transplantation of Teeth, - - - - 575
Unusual Action of Anæsthetics, - - - 522
Unity of Force, - - - - - - 348
Use of Tobacco by Students, - - - - 236
Use of Salicylic Acid in the Household, - - - 35
Use of Cohesive Gold Foil and the Mallet, - - 22
Virginia State Dental Association, - 392, 433, 443
Von Heyden, Dr., - - - - - 35
Vulcanite Litigation, - - - - - 8
Webb, Marshall, H., D. D. S., - - - 107
What Anæsthetic Shall We Use? - - 505, 529
Waterman, S., M. D., - - - - 481, 518

### ARTICLE III.

*The Susquehanna Dental Association.*

The 13th Annual Convention of this Society will be held at the National Hotel, Shamokin, Pa., on Wednesday, May, 16th, 1877, at 10 A. M. Session to continue two days.

Dr. G. T. Barker, of Philadelphia, will give a clinic for the benefit of the Association, which will be very interesting and instructive.

Subjects for Discussion: "Extracting during the Catamenial Period and the Period of Gestation," and "Dental Nutrition." Essayists: Drs. E. B. Long, W. H. Hertz and G. W. Klump.

Others have also kindly volunteered to assist in making the meetings interesting and instructive. All sessions open to the public.

É. B. LONG, D. D. S., *President.* J. H. JOHNSTON, D. D. S., *Rec. Secretary.*

### TO READERS AND CORRESPONDENTS.

Communications intended for publication, and exchange journals and papers, should be addressed to the EDITOR OF THE AMERICAN JOURNAL OF DENTAL SCIENCE, 259 N. Eutaw St., Baltimore, Md. All letters relating to the business of the journal, or containing cash remittances, to be directed to Messrs. SNOWDEN & COWMAN. Articles for publication should be received by the editor at least three weeks previous to the first day of the month on which the number in which it is designed for them to appear, is issued.

### CONTENTS:

ARTICLE I.—The American Academy of Dental Surgery ........ 1

ARTICLE II.—Death of Dr. Chas. A. Jourdan from Effects of Chloroform ........ 6

ARTICLE III—The Susquehanna Dental Association ........ 7

ARTICLE IV.—Vulcanite Litigation ........ 8

ARTICLE V.—Concerning the Causes of Failures in the use of Cohesive Gold Foil; the Mallet, Etc ........ 22

ARTICLE VI.—On Congestion of the Pulp. By Prof. Geo. T. Barker, D. D. S ........ 31

ARTICLE VII.—The Use of Salicylic Acid in the Household. By Dr. Von Heyden, 35

ARTICLE VIII.—Adenoma of Soft Palate ........ 37

ARTICLE IX—Atmospheric Anæsthesia. By M. J. Eley, M. D ........ 39

ARTICLE X.—Possible Dangers from Salicylic Acid ........ 39

### EDITORIAL, ETC.

Comparative Anatomy ........ 40

### BIBLIOGRAPHICAL.

Taft's Operative Dentistry, Third Edition ........ 44

### OBITUARY.

Dr. William D. Jenks ........ 45

### MONTHLY SUMMARY.

Dislocation of the Malar Bone ........ 46

A Secret Pile Remedy ........ 46

Local Action of Astringents upon the Vessels ........ 47

A Young Mother ........ 48

Therapeutic Action of Carbolized Camphor ........ 48

☞The American Journal of Dental Science will contain fifty pages of reading matter in each number, making a volume of about

*Six Hundred* pages

EXCLUSIVE OF ADVERTISEMENTS

THE

### AMERICAN JOURNAL

OF

### DENTAL SCIENCE.

**F. J. S. GORGAS, M. D., D. D. S., Editor.**

---

The *Journal* is issued on the first of each month and contains not less than *fifty* pages of reading matter in each number, exclusive of advertisements, making a volume of *six hundred* pages per annum.

In each number will be found Original Communications, Correspondence, Selected Articles, Monthly Summary, Bibliographical Notices and Editorial Matter, and every effort will be exerted to render the *Journal* worthy of the patronage of the Dental profession.

A generous co-operation is respectfully solicited from the members of the profession; and as the *Journal* has now a printing office of its own, which materially lessens the cost of its publication, it will be issued at the reduced subscription price of

**$2.50 Per Annum in Advance.**

Communications are respectfully solicited on all subjects pertaining to the practice of dentistry, as well as reports of cases occurring in dental practice.

---

### RATES OF ADVERTISING.

| | 1 MO. | 2 MO. | 3 MO. | 6 MO. | 12 MO. |
|---|---|---|---|---|---|
| 1 PAGE. | $15 00 | $22 50 | $30 00 | $50 00 | $100 00 |
| ½ " | 10 00 | 15 00 | 20 00 | 30 00 | 50 00 |
| ¼ " | 7 00 | 11 00 | 15 00 | 20 00 | 30 00 |
| ⅛ " | 5 00 | 8 00 | 11 00 | 15 00 | 20 00 |

All original communications designed for publication, works for review, new instruments for notice, exchanges, &c., should be directed to the editor, 259 N. Eutaw St., Baltimore, Md.

All advertisements and subscriptions should be addressed to

SNOWDEN & COWMAN,
*Publishers of the Am. Journal of Dental Science.*
86 W. FAYETTE ST. BET. CHARLES & LIBERTY STS.,
BALTIMORE, MD.

